# Application and Challenges of IoT Healthcare System in COVID-19

**DOI:** 10.3390/s22197304

**Published:** 2022-09-26

**Authors:** Abdullah A. Al-Atawi, Faheem Khan, Cheong Ghil Kim

**Affiliations:** 1 Department of Computer Science, Applied College, University of Tabuk, Tabuk 47512, Saudi Arabia; 2 Department of Computer Engineering, Gachon University, Seongnam 1342, Korea; 3 Department of Computer Science, Namseoul University, Cheonan 31020, Korea

**Keywords:** COVID-19, healthcare system, internet of things, IoT application

## Abstract

The importance of the IoT is increasing in every field of life, and it especially has a significant role in improving the efficiency of the healthcare system. Its demand further increased during COVID-19 to facilitate the patient remotely from their home digitally. Every time the COVID-19 patient visited the doctor for minor complications, it increased the risk of spreading the virus and the cost for the patient. Another alarming situation arose when a patient was in a critical position and may not claim an emergency service from the nearby healthcare system, increasing the death rate. The IoT uses healthcare services to properly monitor COVID-19 patients by using the interconnected network to overcome these issues. Through the IoT, the patient is facilitated by the health care system without spreading the virus, decreasing the death ratio during COVID-19. This paper aims to discuss different applications, technologies, and challenges of the IoT healthcare system, related to COVID-19. Different databases were searched using keywords in PubMed, ResearchGate, Scopus, ACM, Springer, Elsevier, Google Scholar, etc. This paper is trying to discuss, identify, and highlight the useful applications of the IoT healthcare system to provide guidelines to the researchers, healthcare institutions, and scientists to overcomes the hazards of COVID-19 pandemics. Hence, IoT is beneficial by identifying the symptoms of COVID-19 patients and by providing better treatments that use the healthcare system efficiently. At the end of the paper, challenges and future work are discussed, along with useful suggestions through which scientists can benefit from the IoT healthcare system during COVID-19 and in a severe pandemic. The survey paper is not limited to the healthcare system and COVID-19, but it can be beneficial for future pandemics or in a worse situation.

## 1. Introduction

The coronavirus is officially named COVID-19 by the World Health Organization (WHO). COVID-19 is a severe concern for every field of life, including political, public health, religion, economics, culture, education system, etc. The coronavirus is a lethal respiratory and influenza infection in humans, and the total infected cases are 152 million, recovered patients are 89.9 million, and the deaths are 3.19 million until 2 May 2021. The statistics show the severity of the disease, and the researchers are doing their best to find the proper solution [1,2]. The treatment is essential in COVID-19, along with the precautions and care, and it can be successfully implemented by using the Internet of Things (IoT) [3,4,5,6,7]. The IoT and advanced communication [8,9] is a well-defined scheme of the interconnection of physical objects or things such as computing, digital, and mechanical devices embedded with sensors to gather information between each other with less human intervention.

The IoT is very useful in accessing and controlling things remotely within the existing infrastructure. This generates an opportunity for embedding digital systems and physical objects as well as increases social, technical, and economic benefits. The physical objects range from a small nanochip to a large-size router or communication antenna. These things are used in combination with actuators, sensors, and software to transmit and receive data packets. The IoT has a widespread application, and the connection of things is increasing exponentially. It is stated that after 2020, the number of things will exceed the total population of the world. Likewise, the investment in IoT in 2019 was USD 7.7 billion, and it is expected that it will exceed a value of 22.36 billion by 2025 [10].

**Figure 1:**
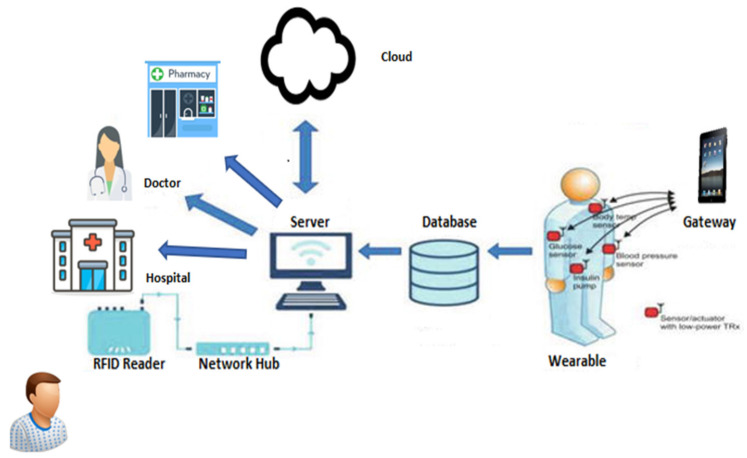
The IoT Network.

Over the past few years, many healthcare applications [11,12,13,14,15] related to IoT have been suggested to ease patients, doctors, and administration. The IoT applications improve the existing healthcare system by offering real-time monitoring regarding the patient’s condition, medical emergency management, etc. These applications enable the patient to record and monitor their current health condition related to blood pressure, sugar level, heart functioning, physical fitness, etc. in the form of data and forward it to the concerned health center or doctor. Healthcare centers or hospitals are also using the IoT applications widely, and they provide real-time services by finding their location and health conditions, as shown in Figure 1.

The world and the research community are using the IoT applications with real-time connected devices to bring improvement in daily life. The IoT is gaining its reputation in every field of life, especially in the healthcare system, by providing clinical services, monitoring of the patient, advanced detection and monitoring of medical problems, computer-based rehabilitation, and reliable emergency services to the coronavirus patient during COVID-19 [16]. The smartphone enables all the activities quickly by monitoring and collecting the updates of the patient regularly [17]. The monitoring and updates are possible with the help of sensors that are implemented and installed in the wards, diagnostic instruments, and emergency services to periodically communicate the data to the nearby health care system [18] for rapid response. With the help of the IoT, the healthcare system is offering an efficient and reliable monitoring and tracking system to the resource management of the community [19]. Similarly, cloud computing plays its role through data storage, sharing of resources, integration of data service, and early security warning to the healthcare system [20]. Likewise, this paper [21] explains the adaptation and application of the IoT in healthcare system through systematic review. This paper is a state-of-the-art survey, but important discussion is related to deep learning for the IoT, data collection method from different databases, the characteristics of the collected data, testing and tracing of COVID through IoT, and regulation for IoT during pandemic. However, in the characteristics this paper has very limited information about the technology, application, and architecture of the IoT during the pandemic. However, in the proposed paper technology, application and architecture of the IoT during pandemics are explained with more details, diagrams, and explanation along with suggested challenges.

### 1.1. Contribution and Scope

The survey is presenting state-of-the-art methods related to the IoT healthcare-based systems in COVID-19. This survey also highlights the work completed in the IoT healthcare system, but the primary focus of this paper is to benefit the healthcare system from the IoT during the pandemic of COVID-19. Furthermore, this paper aims to give new directions to the researcher community in developing more advanced and user-friendly IoT applications during the COVID-19.

The paper is divided into four section to supplement the innovation of the Internet of things in the health care system during COVID-19, i.e., Architecture of the IoT during COVID-19, applications of the IoT during COVID-19, utilization of smart technologies during COVID-19, and finally the challenges of the IoT during COVID-19. This paper proposed that if the architecture, application, and smart technologies were properly used, then the challenges could be reduced during COVID-19 and future pandemics.

To understand the IoT services, it is essential to understand the architecture and elements of the IoT. Therefore, a thorough literature review was conducted and only those IoT healthcare applications were included in this paper, which could facilitate the COVID-19 patient during the pandemic.

In this paper, many important IoT applications are discussed for the healthcare system to increase patient care, enhance the workflow, efficient utilization of the limited resources, significant cost saving of the patient, etc. Additionally, different technologies are highlighted in this paper, and recommendations are given about using various techniques in different situations, patients, disease, location, etc.

Finally, many challenges and impacts during COVID-19 are highlighted related to the IoT healthcare system. However, an improved IoT system has yet to be made to overcome the significant challenges during COVID-19 faced by the healthcare system.

### 1.2. Organization of the Paper

**Figure 2:**
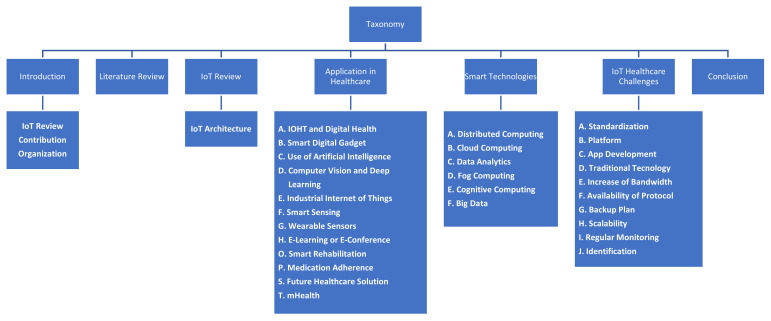
Taxonomy.

The taxonomy shows that this paper is divided into seven sections, as shown in Figure 2.

Section 1 explains the introduction in detail as well as the overview and background of the IoT healthcare system. Additionally, the contribution and scope of the paper are defined with explicit information. Section 2 describes the literature work related to the IoT healthcare systems and the IoT healthcare systems during COVID-19. Section 3 describes the IoT to better understand the healthcare system during COVID-19 and then further divided into architecture and elements. Section 4 is the most essential part of the paper, including almost all the relevant applications related to the IoT healthcare system during COVID-19. The healthcare system uses all these applications, and many of them are used for COVID-19. All these applications can be used during COVID-19 and for future pandemics to benefit and prepare society and humans against any kind of pandemics. Section 5 describes the technologies in detail used during COVID-19 and could be used in the future against the COVID-19 and other pandemics. Section 6 describes the challenges and future direction related to the IoT healthcare system in a normal situation and in a pandemic situation that provides more information than previous papers. Section 7 finally concludes the article.

## 2. Literature Work

**Figure 3:**
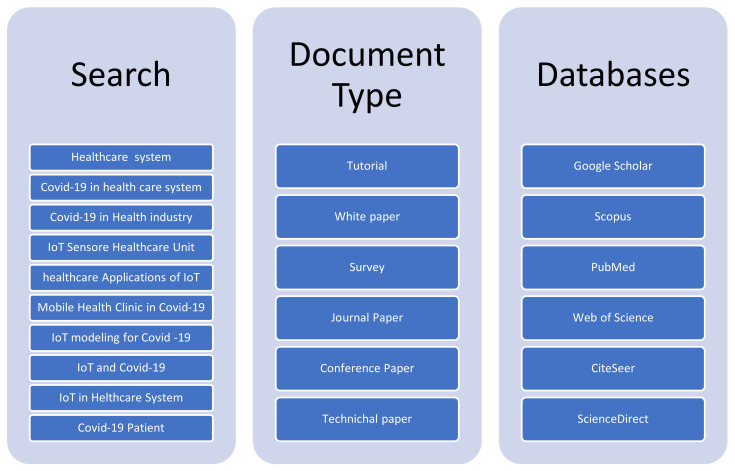
Data Collection Criteria.

The proposed paper examines the chronological order of the healthcare system and the IoT in parallel. In primary reviews, many articles that were collected were related to the healthcare system, but in the secondary review, those papers related to the healthcare system during COVID-19 in the IoT environment. The search term was performed using different IoT keywords with the health care system and COVID-19 category. For search terms, various databases and document types were searched, which is shown in Figure 3.

During the pandemic situation, the number of COVID-19 patients is increasing rapidly around the globe, and now it is compulsory to implement a well-organized and systematic IoT methodology. The IoT is already providing services in many other fields and fulfilling its purposes such as the Internet of Healthcare Things (IoHT) and the Internet of Medical Things (IoMT). To follow the guidelines and facilities offered by IoHT and IoMT, the COVID-19 pandemic can be controlled and minimized.

In [22,23], cloud computing is used in parallel with the IoT for the healthcare system. In the former paper, the IoT applications are used in a semantic approach to deal with the challenges and then proposed a cloud-based model to offer a set of functionalities to diagnose a patient remotely. In a later approach, an android application in a healthcare system is implemented using cloud computing and IoT. In [24,25], the researcher designed a new tool for the healthcare system due to the increase in the IoT demand. In both papers, the patient was monitored through his heart rate value. In the former, it was achieved through a smart health band, and the patient’s information was communicated to his friends and family members. In the latter approach, the heart patient was informed through an early detection technique known as iCarMa. Early detection and diagnosis of the patient’s condition were communicated in this technique, and many precious lives were saved. Currently, the IoT has become the most essential part of the medical care system, and patient information should be kept safe from unauthorized users.

All the papers mentioned above are beneficial for detecting patient history or condition and then transferring data to the healthcare system and emergency system to facilitate the patient. In the proposed paper, all the practices were entertained for the patient through the IoT. Suggestions are given about the healthcare system for tackling the patient since the threat level was more severe in a heart patient or diabetic patient. This paper surveys the new and updated IoT-based healthcare applications and technologies during this worse pandemic scenario, where the number of infected patients was increasing every day worldwide. However, the IoT was still a novel approach for many healthcare professionals, but its implementation was inevitable in the healthcare system due to its cost-effectiveness and practical solution.

## 3. IoT Overview

### 3.1. IoT Architecture

**Figure 4:**
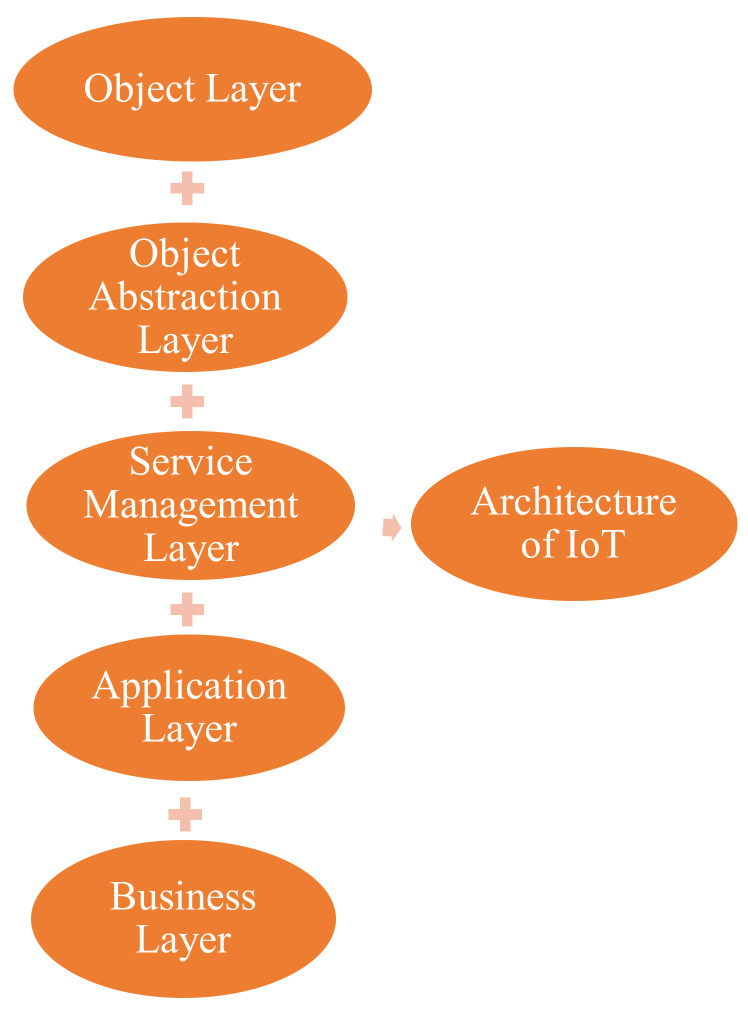
Architecture of IoT.

The ability of the Internet of Things is the interconnection of heterogenous objects through the internet, and it is very necessary to design a flexible layered architecture. There are also some projects such as the IoT-A [26] that are designing a general architecture on the requirement of the industry and researcher. According to the literature survey, the basic architecture is a 3-layer model [27,28,29] consisting of a perception layer, network layer, and application layer. Likewise, some other models have been suggested enhancing more abstraction to the Internet of Things architecture [30,31,32]. There are some common IoT architecture, i.e., three-layer based architecture, middle-ware based architecture, SOA based architecture, and five-layer based architectures. However, in this paper five-layer based architectures are discussed along with each layer in detail, as shown in Figure 4.

Three-layer based architectures were designed when the connected IoT devices were limited. However, with the introduction of the IoT in the healthcare system, we shifted to the 5-layer architectures by adding a service management layer and a business layer. The perception layer will allow us for data storage in the form of big data or the cloud, as well as it takes the decision about the patient data. For example, if the sensed data of the patient was critical, then it would inform and update the patient, healthcare system, and emergency services, which was the most important feature of the 5-layer architecture. Similarly, the business layer is also responsible for implementation, monitoring, designing, analyzing, and developing IoT-related elements.

#### 3.1.1. Object Layer

**Figure 5:**
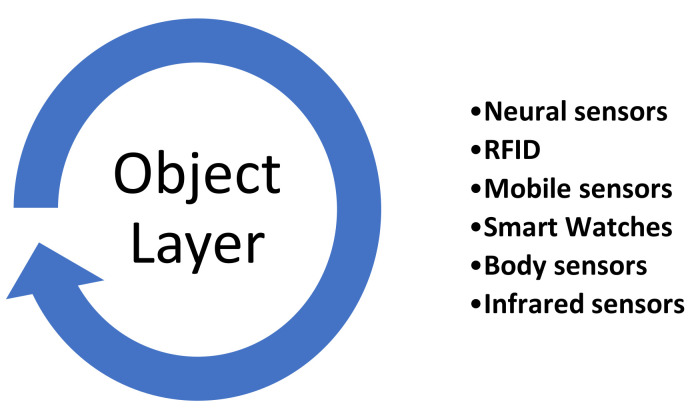
Object Layer.

It is the first layer, and it is used to collect and process the information between the physical objects such as sensors, actuators, and other devices as shown in Figure 5. These physical objects are used for multiple purposes such as temperature sensing, vibration, motion, querying location, etc. A proper mechanism is used for data transferring through a secure channel by the object layer to configure heterogenous objects. The large amount of data sensing and the production of digitized data is accomplished through the object layer [28,29].

Currently, smart devices such as sensors and actuators are smaller, adaptable, multipurpose, and power efficient. Multiple platform and services are used for the integration of these devices and the app developers were provided an access through these platforms, which is known as interoperability. The interoperability is implemented device to device communication between smart phones, watches, and wearable devices.

Multiple communication protocols of the IoT are implemented at the object abstraction layer using cellular, Wi-Fi, Bluetooth, and NFC. However, traditional protocols such as ZigBee, LoRa, ISA100, etc. are used for data communication between the smart devices. Due to various smart devices, the IoT object cannot be supported by traditional communication and need special solutions such as gateway-based solutions, where a gateway interconnects the smart devices using IPV6 protocol.

The World Health Organization (WHO) used standard medical protocol for the prevention of the obesity [33,34]. This protocol would collect the values of overweight, underweight, and normal weight relevant to their age, diet, medication, height, body mass, and blood pressure. By using the IoT platform, the after-mentioned variables were monitored from the healthcare system during exercise and mobility inside their homes, offices, and outside. This system was developed by the IoT platform named INTER Health [35]. Such type of application was also very useful during the COVID-19 pandemic and could be implemented with minor modifications.

INTER-IoT provided flexibility and improvement by connecting the IoT devices with each other and with the IoT platform. This would allow the device to connect the patient and healthcare system during COVID-19 and would perform the proactive communication regarding the COVID-19 patient condition and movement [36].

#### 3.1.2. Object Abstraction Layer

**Figure 6:**
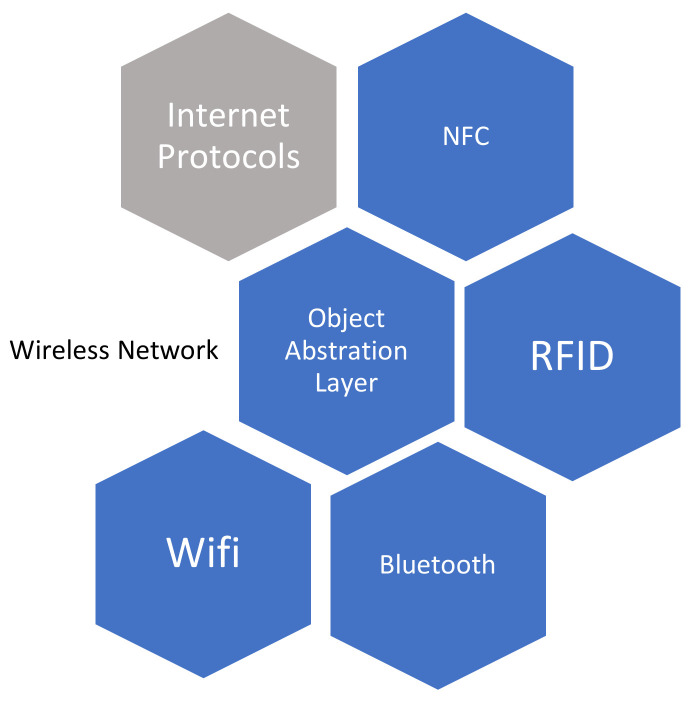
Communication layer.

The object layer is responsible for the production of data, and now it is the responsibility of the object abstraction layer to transfer the produced data between the object layer and service management layer through a secure mechanism. Now various technologies, i.e., Wi-Fi, GSM, 3G, ZigBee, Bluetooth, Near Field Communication, etc. are connected through wireless networks by using internet protocols for smart objects, which are used by the object abstraction layer for data transferring between the two layers as shown in Figure 6. Furthermore, other responsibilities are performed by the object abstraction layer such as the data management process and cloud computing [28]. Some of the applications such as health monitoring, fitness tracking, and emergency services are used for patient information through smart watches, mobiles, and body sensors.

#### 3.1.3. Service Management Layer

This layer is pairing a service with the requester’s name and address. The service management layer is providing a service to the IoT application programmer to coordinate with the objects layer, irrespective of the specific hardware platform. Similarly, the service management layer processes the received data from the object layer and then proceeds with the decision on the basis of the process data. At the end, the process data, along with the decision, are transferred to the desired services over the network wire protocols [29,32,37].

#### 3.1.4. Application Layer

This layer provides the services on demand and on the request of the user. For example, a researcher, students, or customers need the data of the air humidity and air temperature. Therefore, it is the responsibility of the application layer to provide the data as per the demands of the user. This layer is providing services to smart homes, smart cities, smart transportation, smart businesses, healthcare systems, etc. Furthermore, the IoT with the application layer improves the services by providing quality services, according to the user demand, which improves the social, financial, and technical aspects for the application layer [28,37].

The responsibility of application layer is data formatting and the presentation in traditional network. Traditional networking is based on HTTP, but HTTP is not suitable due to larger overhead in resource limited conditions. Therefore, other protocols are used in the IoT environments such as MQTT (Message Queue Telemetry Transport) and CoAP (Constrained Application Protocol).

#### 3.1.5. Business Layer

**Figure 7:**
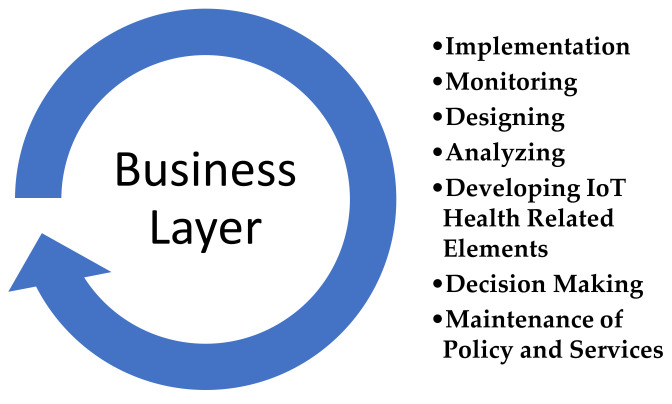
Business Layer.

This layer manages the overall activities and services of the IoT in a healthcare system. This layer receives all the data from the application layer and on the basis of that information the business layer tries to build a graph, business model, flowcharts, etc. Likewise, this layer is responsible for the implementation, monitoring, designing, analyzing, developing IoT-related elements, and decision-making process related to big data analysis, as shown in Figure 7. Furthermore, it is the responsibility of this layer to maintain and monitor the underlaying four layers. This layer is also maintaining the user policy and improving the services by comparing the output of each layer with the expected output [29,37].

## 4. Applications of the IoT HealthCare System during COVID-19

**Figure 8:**
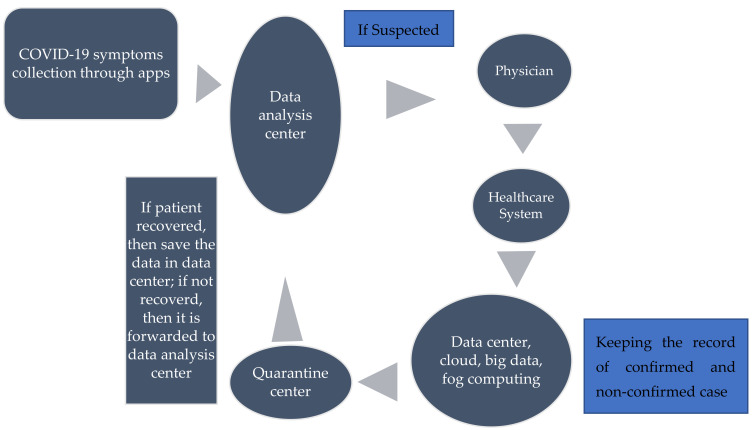
Structure diagram of the IOT healthcare system during COVID-19.

In the IoT of the healthcare system, services and applications are very compulsory for each other and applications are proposed on the demand of the user and customer, therefore, it is directly related to the user or patient requirement. Hence, applications are user-centric, and services are developer-centric since services are used to develop applications. Therefore, the integration of technology has enabled the IoT to be one of the reliable technologies during COVID-19, and it plays a crucial role in reducing the outbreak of COVID-19 [38]. First, the apps are used for the detection of the COVID-19 symptoms and forwarded to the data analysis center about the decision of COVID-19 symptoms. If the patient is detected with the symptoms, then the patient is referred to the physician. The physician contacts the healthcare system and the healthcare system saves the data in the data center. In the data center, different technologies are used, as discussed in Section 6. After saving the information of the patient, it is transferred to the quarantine center for further treatment. If the patient has still not recovered, then again, his data is forwarded to the data analysis center for further decision. It should be noted whether the patient is recovered or not, his data is stored in the data center as shown in Figure 8. In this section, various applications and their services are discussed for different healthcare solutions during COVID-19, as shown in Table 1.

### 4.1. Internet of Health Things and Digital Telehealth

It is an extended version of IoT, which aims to monitor and communicate patient conditions to the healthcare system through wired and wireless communication [39,40]. Nowadays, telemedicine is a popular method to provide the services of an experienced physician to a patient living in a remote area [41].

Let’s consider four patients, i.e., A, B, C, and D. Patient A needs an evaluation of mild respiratory symptoms but is restricted to going to the hospital. Patient B has no signs of the virus but is in contact with the coronavirus patient and wants to be evaluated. Patient C is a coronavirus patient and wants to be checked regularly from home. Patient D is hospitalized in a remote location and wants to consult with a more experienced physician. There are many situations during COVID-19 in which patients and service providers are using telemedicine, while maintaining social distancing. The data of the patients are collected and transmitted to the cloud through a local gateway. The doctor checks the patient information using a digital application and prescribes the guidelines related to the COVID-19 patient to medical staff, as well as a friend or family member of the patient [39]. Many countries began telemedicine during COVID-19 to prevent the outbreak. Many applications like Health Arc [42], Health Net connect [43], Sehatyab [44], Continuous Care [45], etc. are facilitating COVID-19 patients through telemedicine.

### 4.2. Smart Digital Gadget

These digital gadgets are very useful for the self-monitoring of health. The patient can self-monitor their temperature, heartbeat, exercise, nutrition, etc. by using these gadgets. The patient can see their report through these gadgets and forward the report to the concerned physician for necessary treatments. These gadgets can also be very practical during the COVID-19 pandemic and can be modified further for the COVID-19 situation. These gadgets can communicate real-time information related to the presence of an infected patient, the danger zone declared by the government related to the coronavirus outbreak, violation of social distancing, identification of nearby healthcare center of COVID-19 patients, etc. IoT-based ventilators and temperature monitors are compulsory for COVID-19 patients, and these can provide timely assistance that is helpful for patient recovery. These gadgets can also activate the alarm if the protocols of COVID-19 are not followed [46].

### 4.3. Industrial Internet of Things (IIoT)

It comprises sensors, actuators, and other connected devices connected with industrial computer applications for energy management and manufacturing [47]. The connectivity provides data collection, transmission, and analysis, which increases the efficiency of energy management and manufacturing if working from a remote location and, as a result, increases the economic benefit of the industry. Hence, IIoT, similar to serious games [48,49] in COVID-19, can play an active role by maintaining the industry’s economic growth and the country.

### 4.4. Smart Sensing

The automatic body temperature sensors and face detection methods are installed in many countries, such as Korea and Japan. The camera and sensors are connected to the server and the cloud. With the help of the temperature sensor, the patient is detected. The patient is identified using a face detector since with face detection, all his information is stored in the centralized database. Smart cities include smart universities, campuses, smart homes, smart markets, etc. AI technology has used to transfers real-time data to the concerned authorities [50].

### 4.5. Wearable Sensors

This enables the hospital to operate virtually along with the detection and prevention of the disease. These devices are lightweight and can monitor almost all of the body’s vital signs, which is forwarded to the medical professional for further process and early detection of the disease. The Philips company in May 2020 developed its wearable biosensor that could continuously monitor the respiratory rate and heart rate. Likewise, kinsa invented the app-enabled smart thermometer in COVID-19 that shows the outbreak in a specified location. A smart inhaler connects with the mobile app through Bluetooth or Wi-Fi. The inhaler is associated with the IoT through various sensors, which transfers the patient data to the concerned physician with the location and time. The world’s first smart inhaler which uses real-time data is Tevas ProAir Digihaler and is connected through the mobile app. In COVID-19, this smart inhaler is very useful by showing the patient’s time, location, and condition because the patient suffering from asthma is more vulnerable to COVID-19 [51,52].

### 4.6. E-Learning and E-Conferencing

During COVID-19, all the educational institutions stopped their academic activities, and the students and teachers were instructed to take their online classes from home. Even most corporate sectors advised their employees to work from home and take the conferences and meetings online from home. The need for E-learning and E-conference was never before, and therefore, it required digital state of the art infrastructure, devices and internet connectivity. Multiple platforms like google classroom [53], zoom [54], Microsoft teams [55] and Skype [56] are used by the institution and corporate sectors. The IoT plays a significant role between smart infrastructure and the platform mentioned above, and successfully provides the whole operation during this pandemic and can be further improved.

### 4.7. Smart Rehabilitation

COVID-19 brings hazards, and many deaths occur due to Severe Acute Respiratory Syndrome Coronavirus 2 (SARS-CoV-2). SARS-CoV-2 surprised the healthcare system by increasing the number of COVID-19 patients in a short time [57,58]. COVID-19 causes respiratory and interstitial pneumonia disorder that in many causes can cause the death of a patient. Furthermore, it causes the failure of kidneys, heart, and vascular muscles [59]. After this critical phase, the patient should be provided with a rehabilitation system to restart their own traditional life. After the disaster caused by COVID-19 in Italy, the San Raffaele Scientific Institute started the rehabilitation center for 50 patients of COVID-19. In the rehabilitation center, the patient’s nutrition, progress, and medication were monitored by the medical teams continuously. Multiple IoT rehabilitation centers exist for the hemiplegic patient [60], smart hospitals, and wards during COVID-19, dedicated health care system for COVID-19, smart city [61] rehabilitation system, etc.

### 4.8. Medication Adherence

The denial and carelessness with medications are serious issues for the patient, patient family, physician, and healthcare system. In today’s world, there are many activities and responsibilities for every individual. It is challenging for them to follow the medication prescribed by the doctors, especially in aged people. Additionally, due to the increase in medication prices, they do not follow the doctor’s prescription. Many types of research are continuing by monitoring the patient submission to medication through the IoT [62,63,64,65]. This [66] paper developed a method that reminds the patient about their medication and measures blood pressure, sugar level, ECG, and temperature. The information is stored on the cloud or dedicated server for the concerned physicians and the medical care unit as a patient’s history. This data is used for further medication by the doctor. The medical care stem also uses the medication system through the IoT to confront COVID-19 [67,68,69].

### 4.9. Mobile Health (mHealth)

**Figure 9:**
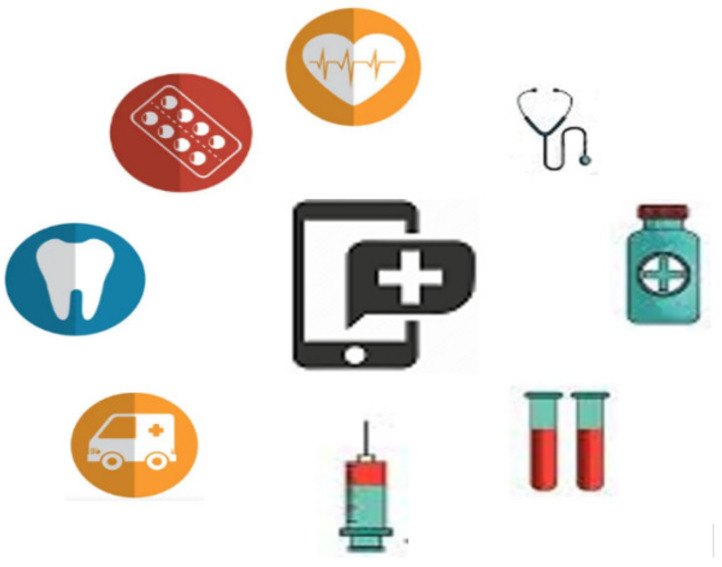
mHealth.

mHealth is used for the sharing and monitoring of patient data through mobile technology and apps, as shown in Figure 9. This data is shared with the concerned physician and healthcare system to identify the symptoms more efficiently (quickly) and reliably (less error and complications). Nowadays, the user minimizes its dependency entirely on the physicians and embraces a mobile healthcare technology in order to be aware of its health data in real-time. mHealth is gaining popularity in consumers and the healthcare industry, as there are more than 318,000 mHealth care apps available in the Google and Apple app stores as of November 2017. Some of the most used apps are diabetes, heart rate, weight loss, blood pressure, pregnant women, aged people, etc.

In recent years, especially during COVID-19, smartphone and healthcare apps have become an integrated part of the IoT. Much research is making a smartphone a multipurpose device, especially in the healthcare system. A smartphone and its related apps must be user-friendly and easily understandable by the patient and the physician [70,71,72,73,74,75].

There are many advantages of smartphone healthcare applications, such as saving time and money, but there are many other challenges, such as energy consumption, loud environments, and computational complexity surrounding the smart device. Nevertheless, smart mobiles effectively use smart healthcare-based solutions that encourage the researcher to use them in the COVID-19 and future pandemic.

### 4.10. Apps Related to COVID-19 by Different Countries

The COVID-19 app is becoming famous and developed at a rapid speed by different companies in different countries; in India a COVID-19 tacking app such as Aarogya Setu was developed during COVID-19. The Aarogya Setu app was developed to track the COVID-19 patient in the ongoing pandemic. The app functions on Bluetooth and GPS to inform the user about the nearby COVID-19 infected patient. The app uses various concepts of artificial intelligence and computer vision to detect and isolate the virus [76].

Similarly, some other tracing techniques such as contact tracing and case isolation are used during the pandemic [77]. The former process [78] usually uses recalling and listing of all individuals with whom the COVID-19 patient makes any contact. The identified individuals in the list are asked to self-quarantine themselves for 14 days or more. This app uses GPS, Bluetooth, and Application Program Interface (API) based technologies. With the help of GPS, the patient location is tracked along with those people who spent time on the same location and at the same time. In Bluetooth, the exchanges are performed through Bluetooth within the close proximity and this technique is considered to be the most suitable for preserving the privacy of the user [79]. In the API based approach, Android and iOS use API by Google and Apple to communicate between the devices through Bluetooth. However, the security concern still remains and to resolve the privacy issue of the user, a privacy-preserving app called Ketzu was designed in Finland. The following apps are shown in Table 2, and designed by different countries for different purposes during COVID-19 [80].

## 5. Smart Technologies of the IoT Based Healthcare System for COVID-19

Nowadays, many technologies for the IoT are used by the healthcare system. The use of the IoT technologies is increasing very rapidly due to COVID-19. Many new technologies by the IoT are introduced over time, according to the demand of the user and industry. Some of the technologies that are and could be very useful for future IoT healthcare systems are as follows:

### 5.1. Mobile Health (mHealth) Distributed Computing

During a pandemic such as COVID-19, the concept of grid computing and distributed computing is introduced since the clusters cannot handle a sufficient amount of computation for a medical sensor. Grid computing and distributed computing are collaborative and decentralized in pattern, and the user does not pay for the use [81]. In traditional computing, the cluster cannot collect this massive amount of data during COVID-19 because the data of the entire area or city should be required. For example, during the worst situation of COVID-19 in India [81], a lot of data was necessary since the population of India is more than 1.3 billion. Then the number of sensors used for this massive amount of data would also increase, and hence increases the computation of data. Therefore, the integration of the IoT with grid and distributed computing is very compulsory during pandemics.

### 5.2. Cloud Computing

Cloud computing is a centralized executive and, therefore, more scalable and flexible than grid computing. Cloud computing [82] is a platform that provides services to grid computing and providing access to the shared resources. The cloud processes the data received from the grid during COVID-19 and then executes an operation to facilitate the patient and the healthcare system.

### 5.3. Data Analytics

In data analysis, data is evaluated and transformed, and some useful information of interest is collected. This information will benefit the decision-making process, which is fast and efficient. In data analysis, the data is extracted from the cloud through the IoT [83]. In the COVID-19 situation, all the information is stored in the cloud. Therefore, it will be advantageous to use data analytics for the patient, doctors, and the healthcare system.

### 5.4. Fog Computing

It is decentralized computing, where computing resources reside between the cloud and the data center. Fog computing is providing on-demand services at the edge network. Fog computing offers high security, high computing data center [84,85], and low latency as compared to cloud computing. The wireless communication [86] supported by fog computing is Wi-Fi, 3G, 4G, Zigbee, Mobile ad-hoc network [87,88,89], and wired communication. Hence, it can efficiently work in a pandemic situation for information exchange between the patient, emergency services, and healthcare system, especially for the patients in the remote area.

### 5.5. Cognitive Computing

It is the process through which computerized models simulated the human assumption/idea in a complex situation, and the results may be undefined. Sensor devices and AI are advancing every day, and the integration of the IoT can mimic human thoughts in solving problems. Therefore, it will be possible to analyze the hidden data in a cloud and increase the ability of the sensors to proceed with the healthcare data and take the decision related to the data automatically. Hence, during COVID-19, the patient’s critical data can be monitored, recorded, and analyzed through cognitive computing [90].

### 5.6. Big Data

Big data means data of a huge size, i.e., an extensive collection of data and increases exponentially with time [91]. Healthcare organizations use big data of a specific population or patient to improve clinical outcomes, reduce cost, provide more targeted healthcare results to clinics and hospitals, increasing advancement in the cure and research, prevent the onset of diseases, and increase the efficiency of the healthcare departments. Through big data [92] electronic healthcare records, patient history, patient lab test, pharmaceutical data, telemedicine, clinic visits, etc. Hence, the IoT [93,94] integration with big data can facilitate the COVID-19 patient in a more accurate and improved way and try to detect and minimize the pandemic.

## 6. IoT Based Healthcare Challenges during COVID-19

Due to the increase in the demand from different industries in the IoT healthcare system, many researchers are working on designing and implementing IoT-based healthcare applications. However, there are many issues in IoT-based applications, which are very significant and need special attention to improve IoT applications and services, especially in the healthcare system. Therefore, many databases like IEEE, ACM, Google Scholar, Willey, Elsevier, Springer, Scopus, etc., are searched for the most recent challenges in the IoT within the field of healthcare. To overcome these challenges, the IoT will reduce the technical gap between patients, doctors, caregivers, and the healthcare system.

### 6.1. Standardization

The IoT is still new in computer science and IT; therefore, many vendors do not follow specific standards regarding interface compatibility and protocols and therefore, make interoperability between the devices. Similarly, the management of value-added services such as access management and registration of healthcare professionals is another standardization issue. It should be resolved as soon as possible by working together with mHealth, eHealth, IoT organizations, and standardization bodies such as the Information Technology and Innovation Foundation and European Telecommunication Standard Institute, etc.

### 6.2. Platform

The platform of the IoT is different from the IoT-based healthcare hardware, and it needs a real-time operating system and a suitable computing platform. Therefore, this platform method should use Service Oriented Approach (SOA) by using diverse Application Package Interfaces (APIs) with run-time libraries.

### 6.3. App Development

App development is a very technical issue, and apps are designed without physician, patient, caregiver, or healthcare experts. Hence, during any app development, participation of the concerned people and authority should be compulsory to ensure the quality app. Further, the app should be regularly updated if new changes are required or if some new pandemic is introduced to the world.

### 6.4. Traditional Technology

The healthcare system should replace its obsolete devices and sensors to become adoptable with the existing current configuration. However, this will increase the healthcare system’s cost and face the compatibility issue between the IoT system and existing devices.

### 6.5. Increase of Bandwidth

In the existing system, networking devices are only used for operating mobile devices and Desktop PCs to operate the administrative issue of the hospital and some lightweight application. However, by introducing the IoT, thousands of sensors and other related devices, the data transfer of the patient to the servers or clouds is required. When data transmission and communication increase, the healthcare system needs updating and advanced networking devices, i.e., routers, switches, updated cabling, and large databases. Therefore, a large bandwidth must efficiently and reliably continue the communication and transmission process between the patient, physician and healthcare system.

### 6.6. Availability of Protocol

IoT healthcare devices are heterogeneous, and the communication layers can operate in many scenarios such as reception, transmission, deep sleep, sleep, etc. Still, the requirement of each layer is different from the other layer in term of power. Therefore, the situation is challenging when a device discovery protocol confirming the availability of services at the MAC layer with minimum energy is used [95]. Consequently, it is necessary to make a power-efficient protocol at each layer for specific devices and in a particular situation, and it will be more adaptable in pandemic situations.

### 6.7. Backup Plan

In COVID-19, real-time IoT data processing between the patient, doctor, and the healthcare system is a significant challenge. The healthcare system should have its directorate of IT where the patient’s data is saved, where it makes communication possible between the patient, doctor, and the healthcare system. The directorate of IT should have a secondary plan for data storage. In case of hardware failure, software failure, or human failure, the system should run continuously without any disruption. Likewise, the healthcare center should also have a backup plan for electricity failure in batteries, a generator, or a solar system. The backup plans are necessary for the healthcare system because the gap/interval between the patient and the healthcare system is critical in a life-saving situation and could be controlled through a backup plan.

### 6.8. Scalability

To offer more services, adding new devices, apps, and functions according to customer requirement without compromising the quality of service is the main challenge in the IoT healthcare system. Introducing new hardware and communication protocols in the presence of traditional devices is a very tedious task, and it must be functional to allow scalable services and operations. Currently, a research group is working on IoT-iCore3 to design a layered framework for the registration, entity discovery, and lookup, interoperability between the devices and scalable mechanism. This is very important because, in the pandemic situation, the world needs a scalable IoT healthcare system missing in COVID-19.

The scalability is the most important factor that needs to be considered, while designing IoT for the healthcare system. Some of the activities unexpected of the pandemic are becoming more complicated than needing diversity in application due to the increasing number of patients and demand from the healthcare system like COVID-19. The healthcare system was never ready for COVID-19, but scalability is necessary to facilitate the user, patient, physician, and healthcare system in such a pandemic.

### 6.9. Regular Monitoring

Continuous monitoring is essential for the patient’s health, treatment, and care in a normal and abnormal situation within a healthcare system. Therefore, such a system is necessary, which continuously monitors the patient’s health, activity, medication, etc. Furthermore, monitoring the activity (within the home and in society) and medication is essential for COVID-19 patient because they can be the source of the virus and spread the virus within the family members and community.

### 6.10. Identification

In a normal situation, the duties of the caregivers were changing periodically with the patient. However, in the COVID-19 condition, a patient and caregivers should be identified, and the caregivers should be checked appropriately or quarantined for 15 days before assigning them another patient or responsibility. In this way, the virus will not be allowed to spread.

### 6.11. Integration of Medical Records across Borders or Authorizing Different Vaccines

This challenge took prominence during the COVID-19 pandemic since no country in the world was allowed to share the patient data of their national. However, the country shared the summary of the COVID-19 patient without disclosing the identity of the patient. Therefore, many agencies such as Pfizer, Johnson & Johnson, etc. tested the COVID-19 of a traveler if they travelled from one country to another. After testing the individual, the traveler showed their results on their arrival and departure. However, still the challenge remained and the solution for this challenge was the consensus of different countries on some reliable platform, where the data of all the patient can be shared. Some models, such as the Personal Health Record (PHR), are very useful for sharing the patient data. This model was implemented within the country, however if the countries were willing, then they could use such model for sharing patient information by using reliable platforms for using medical records across borders or authorizing different vaccines [96].

### 6.12. Privacy and Security

Security and privacy are the two challenges that are common in the IoT healthcare system due to the continuous increase in the connected devices to the internet. There are many attacks in the IoT, such as Botnet attacks, packet flooding attacks, Slow Loris attacks, jamming attacks, amplification attacks, etc. Likewise, DDoS attacks are increasing significantly during the COVID-19 pandemics, and recently it almost doubles in the healthcare system [97,98,99,100].

Currently, the privacy and security of the IoT is not satisfactory from application level to architecture design and it is necessary to ensure the privacy and security of the patient data in the IoT. Traditional IoT security requirements, such as integrity, availability, authentication, confidentiality, authorization, and access control are not fulfilling the security requirement of the IoT healthcare system because it is only limited to data and now, we need to include “Things”.

It is noticed that more than 90% of the IoT devices are not encrypted and therefore, vulnerable to attacks. Such attacks are not limited to data, but it can create a threat to human life [101]. The IoT in the healthcare system play an important role on patient psychological, physiological, and biological state, as well as threaten patient life. The US food and drug administration has revised the electronic safety procedures and protocols for medical devices, updated from 2018 during current pandemic [102,103].

### 6.13. Future Health Care Solution

The user uses many healthcare applications, but they are still not used efficient during the COVID-19 pandemic. It is expected that soon, and with the growing demand for the IoT, all these devices will be enabled for a pandemic similar to COVID-19. However, some healthcare issues such as hemoglobin detection, abnormalities in cellular growth, oxygen saturation, cancer detection, remote surgery, skin diseases, pneumonia detection, state of the art ECG monitoring, etc. need special attention and require some specific monitoring apps and devices. All these are possible due to the presence of the IoT and smart devices.

## 7. Conclusions

The IoT is the most promising technology for collecting and transferring data over interconnected network without human involvement. This modern technology has a crucial role by providing a platform for the patient to discuss and share health data with the concerned physicians and healthcare system in a real-time. However, there are still many challenges in the IoT related to the healthcare system that need to be resolved to improve the healthcare system.

This survey is related to the performance of the IoT healthcare system in COVID-19. In this survey, it is insisted to use the technology and application of IoT. The survey first discussed the IoT review, in which the architecture and element of IoT were discussed. The paper then explained the current state-of-the-art IoT-related healthcare system for the patient and doctor, especially during COVID-19. More emphasis was given to the researcher that these applications could perform efficiently and play a significant role during COVID-19 and future pandemics.

Similarly, a technology used in IoT and more advanced technology was explained in further details and provided a platform for further research and the IoT healthcare system improvement. Challenges were discussed in detail, and future suggestions would improve the patient’s life and facilitate the physician by removing the deficiencies in the IoT apps and technologies. By fulfilling those challenges, a more advanced IoT healthcare system could be achieved with less cost and comfort to the patient and facilitate the overall healthcare system. As a result, a more progressive healthcare system can be achieved by utilizing the apps and fulfilling the challenges during COVID-19. This paper explained almost all the applications, future technology for the IoT, and related challenges in more detail for the healthcare system, COVID-19, and future pandemics. In the future, Systematic Literature Review (SLR) [104,105] will be used to further explain each and every factor of the IoT related to healthcare system.

## Figures and Tables

**Table 1:** Applications and services.

Application	Target Population	Service
Telemedicine	Remote patient	Monitoring of all diseases
Digital Gadget	Home patient for self-monitoring	Monitoring of all diseases, the identification of an outbreak and the location of virus
Pre-trained Model	Society	For detection, identification, and isolation of patient
IIoT	Industry	Increases energy efficiency, management, and growth of industry
Contact Tracing	Smart cities include smart universities, campuses, smart homes, and smart markets.	Stopping the spreading of disease
Wearable Sensors	Critical patient	Need data in real time
E-learning and E-conference	School, colleges, Universities, offices, etc.	Work from home
Smart Rehabilitation	Critical infected patient	Patient’s nutrition, progress, and medication were monitored
Medication Adherence	Old patient	Medication monitoring
mHealth	All patients	To identify the symptoms more efficiently (quickly) and reliably (less error and complications)

**Table 2:** Apps by different countries.

Country	Mobile APP Name
Austria	Stop Corona
China	Chinese health code Sys
Czech	eRouska
Bahrain	BeAware
Cyprus	CovTracer
Israel	HaMagen
India	Sahyog
Finland	Ketju
Germany	Corona App
France	Stop COVID
Turkey	Hayat Eve Sar
Iran	Mask.ir
UK	NHS COVID-19 App
Italy	Immuni
South Korea	Self-quarantine safety protection
Armenia	COVID-19 Armenia
Canada	Canada COVID-19
USA	Apollo COVID-19
Mexico	COVID-19 Tam
ARGENTINA	CUIDAR COVID-19 ARGENTINA
Chile	cov_cl
Colombia	CoronApp—Colombia
Jamaica	JamCOVID19
Uruguay	Coronavirus UY
Iceland	Rakning C-19
Ireland	patientMpower for COVID-1
Spain	COVID-19.eus
Pakistan	COVID-19 Gov PK
Russia	COVID-19 oнлaйн тecт
Vietnam	FAMILY—COVID 19
Mali	SOS CORONAVIRUS
Saudi Arabia	Tawakkalna

## Data Availability

Not applicable.
